# Risk factors for globe removal after open-globe injury in agricultural regions

**DOI:** 10.1038/s41598-022-21666-7

**Published:** 2022-10-12

**Authors:** Orapan Aryasit, Chayut Tassanasunthornwong, Narisa Rattanalert, Supaporn Tengtrisorn, Penny Singha

**Affiliations:** Department of Ophthalmology, Faculty of Medicine, Prince of Songkla University, 15, Kanjanavanich Road, Kohong, Hat Yai, 90110 Songkhla Thailand

**Keywords:** Diseases, Health care, Health occupations, Medical research, Risk factors, Signs and symptoms

## Abstract

This study aimed to identify the prevalence and risk factors for globe removal among patients with open-globe injuries (OGIs) in agricultural regions. A retrospective chart review of patients with OGIs was performed between January 2010 and December 2019. Univariable and multivariable logistic regression models were used to identify the factors associated with globe removal in OGI. This study included 422 patients (422 eyes). The highest prevalence of OGI was observed in the middle age group (> 20 to 40 years). The most common cause of OGIs was agriculture-related injury (54.7%), followed by industry or workplace-related injury (20.4%), and assault (5.0%). Intraocular foreign bodies, endophthalmitis, and panophthalmitis were reported in 57.6%, 28.4%, and 5.7% of cases, respectively. Eight eviscerations and 43 enucleations were performed, accounting for 12.1% of OGIs. The most common indication for globe removal was panophthalmitis. Multivariable analysis revealed that the predictive factors significantly associated with globe removal were assault injuries (adjusted odds ratio (aOR) = 5.53; *p* = 0.026), presenting logarithm of the minimum angle of resolution visual acuity (aOR = 311.79; *p* < 0.001), and endophthalmitis and panophthalmitis (aOR = 3.58 and 734.94, respectively; *p* < 0.001). This knowledge would aid in patient counseling and encourage health promotion.

## Introduction

Open-globe injury (OGI) is a major cause of permanent visual impairment and blindness. An estimated global incidence rate of 3.5 per 100,000 persons per year, leading to 203,000 OGIs per year, has been reported worldwide^1^. The epidemiology and characteristics of OGI differ depending on the activity at risk in each region^2–4^.

Primary and secondary globe removal from trauma accounted for 40–67% of all patients who underwent the removal of an eye^5,6^. The proportion of globe removal in patients with OGIs in developed countries ranges from 8.3 to 11%^7,8^. However, a higher rate of globe removal, reaching 16.8–25%, was observed during wartime^9,10^. The rate of globe removal in the case of OGI depends on the mechanism of injury, limitations of medical care, and cultural considerations^11^. To the best of our knowledge, no previous studies have examined the risk factors for globe removal after OGI in agricultural regions with contaminated soils, organisms, plants, and animal products. The prevalence of globe removal after OGI may be higher in the agricultural areas than that in other settings due to a higher rate of eye infection, delayed presentation, and severity of the injury. The risk factors for globe removal in patients with OGIs in this special situation merits further investigation.

The aim of this study was to identify the prevalence and risk factors for globe removal among patients with OGIs in the agricultural region of Southern Thailand. The information obtained from this study could provide material for patient counseling, including appropriate management of OGI and its prognosis.

## Methods

A retrospective chart review of all patients with OGIs who visited our hospital between January 1, 2010 and December 31, 2019 was performed. Approval was received from the Human Research Ethics Committee of the Faculty of Medicine, Prince of Songkla University (REC number 63-019-2-4), and the study was conducted in accordance with the principles of the Declaration of Helsinki. The need for informed consent was waived due to the retrospective nature of the study.

The diagnosis of OGI was based on clinical ocular examination defined by full-thickness laceration of the eyeball, which was classified according to the Birmingham Eye Trauma Terminology System (BETT). BETT classifies OGI into four groups (rupture, penetrating, perforating, and intraocular foreign body (IOFB))^12,13^. The patient, with combined perforating globe injury and IOFB, was classified as a case of perforating injury, because the globe perforation is more severe than IOFB alone. Our study included four patients with perforation combined with IOFB; they had a foreign body that caused the globe perforation along with another retained IOFB. The patients who underwent globe removal at other hospitals or had a follow-up period of less than 3 months were excluded from this study. In total, 441 patients presented with OGIs; 19 patients were excluded as their follow-up period was less than 3 months and 3 patients (15.8%) among these underwent globe removal.

Visual acuity (VA) was recorded using the Early Treatment Diabetic Retinopathy Study (ETDRS) chart, categorized into five groups following Ocular Trauma Score (OTS) (≥ 20/40, 20/50–20/200, 19/200—count fingers (CF), hand movement (HM), light perception (LP), and no light perception (NLP)), and then converted to the logarithm of the minimum angle of resolution (logMAR) for analysis. The logMAR values of 1.9, 2.3, 2.7, and 3 denoted CF, HM, LP, and NLP, respectively^14,15^. The presenting VA, relative afferent pupillary defect (RAPD), mechanisms of injury, zone of injury, indications for globe removal, presence of sympathetic ophthalmia (SO), VA at the final visit, and follow-up period were assessed. We categorized the combined zone into the most posterior zone, which represents the most severe injury. OTS was calculated based on the clinical presentation, as described by Kunh et al.^16^. We determined panophthalmitis as endophthalmitis in a variable of OTS.

All data analyses were performed using Stata Statistical Software (Stata/MP 14.1, StataCorp LP, College Station, TX, USA). Descriptive statistics including the frequency, mean, median, and standard deviation (SD) were calculated. Pearson’s chi-square and Fisher’s exact tests were used to analyze the non-parametric data between the globe removal group and the non-globe removal group. The parametric data between the two groups were analyzed using the student’s *t* test. For the bilateral OGIs, one eye was selected to be part of the data analysis using computer program randomization. Univariable logistic regression and multivariable logistic regression models with stepwise backward elimination method were performed. A *p* value of < 0.05 was considered statistically significant. Since the RAPD information was missing for 170 patients, OTS could not be assessed properly. However, to avoid omitting these data altogether, a “reduced OTS” was calculated in which the points for RAPD were not considered. The variable RAPD was considered to have three categories-negative, positive, and missing. To minimize the bias that may result from the incomplete data, the reduced OTS was accompanied by the 3-category RAPD in all subsequent modeling.

### Ethics approval

The study was approved by the Institutional Review Board of the Faculty of Medicine, Prince of Songkla University. The requirement for informed consent was waived by the committee.

## Results

### Demographic data and mechanisms of injuries

Among the 422 patients included in this study, six patients had bilateral OGIs. From these, only one eye was selected for data analysis. Table 1 presents the demographics and clinical details of the injuries. The highest incidence of OGIs was found in males (92.4%). Falls predominantly accounted for OGIs in female patients (4/5, 80%). Assaults accounted for 5.0% of OGIs, predominantly involving adult males (20/21, 95.2%). Agriculture-related injuries accounted for more than half of all patients, with mowing being the major cause (82.7%) (Table 2). Among the 342 referred patients, 148 were referred without primary repair, 145 underwent primary repair at the referral hospital, of which 61 patients required re-suturing at our center, 44 patients had self-sealing wounds, and five patients underwent globe removal. Among the 80 non-referred patients, of which 69 patients underwent primary repair, 10 patients had self-sealing wounds, and one patient underwent globe removal.

**Table 1:** Demographic data and injury types among patients with open globe injuries.

Variables	n (%)
*Gender*	
Male	390 (92.4)
Female	32 (7.6)
*Age, years*	
Mean (SD)	39.04 (17.32)
Median (min, max)	38.39 (2.34, 87.87)
*Age group*	
≤ 20 years	63 (14.9)
> 20 to 40 years	161 (38.2)
> 40 to 60 years	151 (35.8)
> 60 years	47 (11.1)
*Type of injury*	
Accident	401 (95.0)
Assault	21 (5.0)
*Nature of injury*	
Agriculture-related injury	231 (54.7)
Industry or workplace-related injury	86 (20.4)
Recreation-related injury	21 (5.0)
Assault by firearm and explosion	16 (3.8)
Accidental exposure to explosion and firearm	15 (3.6)
Traffic accident	14 (3.3)
Housework-related injury	10 (2.4)
Accident from body part	6 (1.4)
Falling	5 (1.2)
Assault from close combat	5 (1.2)
Others	13 (3.1)

*SD* standard deviation.

**Table 2:** Causes of agriculture-related injury.

Cause of injury	Sex, n (%)		Age (years)
	Male	Female	Mean ± SD
Mowing	187 (84.6)	4 (40.0)	42.7 ± 13.6
Cutting wood	17 (7.7)	3 (30.0)	50.9 ± 19.9
Bird pecking	5 (2.3)	2 (20.0)	39.7 ± 21.7
Agricultural equipment	4 (1.8)	1 (10.0)	62.5 ± 4.4
Others*	8 (3.6)	0 (0.0)	52.0 ± 16.5

*Digging = 2, Bee sting = 1, Coconut falling = 1, Coconut cutting = 1, Fishery = 1, Palm falling = 1, Salak thorn = 1.

*SD* standard deviation.

### Presenting VA and RAPD

The mean VA at presentation for all 422 patients was 1.93 ± 0.91 logMAR VA (CF, ETDRS chart), with 63.7% of patients presenting with VA worse than CF (Table 3). At the final visit, the mean VA was 1.09 ± 1.01 logMAR (16/200, ETDRS chart). Among the patients with NLP at presentation, only one patient reached a VA of LP, while 10 patients remained at a VA of NLP at the final follow-up. The presenting VA of the globe removal group was reported in PL (24 patients), NLP (22 patients), HM (four patients), and CF (one patient). Clear documentation regarding the status of RAPD was available for 252 patients. The presence of RAPD was observed in 52 out of 252 patients. Information regarding RAPD was lacking for 170 patients; therefore, we calculated OTS by excluding RAPD from the multivariable analysis.

**Table 3:** Visual acuity at presentation and at the final follow-up.

Visual acuity	Presenting, n (%)	Final, n (%)
20/40 or better	49 (11.6)	126 (29.9)
20/50 to 20/200	46 (10.9)	107 (25.4)
19/200 to CF	58 (13.7)	42 (10.0)
HM to LP	236 (55.9)	65 (15.4)
NLP	33 (7.8)	31 (7.3)
Globe removal	NA	51 (12.1)

*n* number of patients; *CF* count fingers; *HM* hand movement; *LP* light perception; *NLP* no light perception; *NA* not available.

### Globe infection and micro-organisms

Out of 422 patients, 144 (34.1%) had endophthalmitis and panophthalmitis. Nine out of 80 non-referred patients had endophthalmitis. Among the 342 referred patients, 111 had endophthalmitis, and 24 had panophthalmitis. Excluding the patients with panophthalmitis, 210 patients had delayed presentation (visit hospital after injuries more than 24 h), of which 77 patients (36.7%) were diagnosed with endophthalmitis. Out of the 188 patients who visited the hospital within 24 h, 43 patients (22.9%) developed endophthalmitis. Delayed presentation was significantly associated with endophthalmitis (*p* = 0.003). The microbial culture was positive in 103 patients: a single species of bacteria was found in 66 patients, multiple species of bacteria in 18 patients, multiple species of bacteria with fungi in 11 patients, and fungi in eight patients. The most commonly organisms in endophthalmitis and panophthalmitis were *Staphylococcus* and *Bacillus* species. *Staphylococcus* species accounted for 37 positive cultures, of which five eyes with positive culture underwent globe removal. *Bacillus* species accounted for 29 positive cultures, of which 14 patients underwent globe removal. *Pseudomonas aeruginosa* accounted for five positive cultures, of which two eyes underwent globe removal.

### Globe removal surgeries

Six out of these 51 patients underwent primary globe removal (five enucleations and one evisceration) with a median time of 12.0 h (interquartile range 7.2 h to 1 day) between the onset of injury and time of globe removal. The indications for primary globe removal were severe disfigurements in four patients and panophthalmitis in two others. Secondary globe removal was performed in 45 patients (38 enucleations and seven eviscerations), with a median time of 4 days (interquartile range 3–15 days) from injury to globe removal. The cause of secondary globe removal was panophthalmitis in 21 patients, intractable endophthalmitis in nine patients, severe disfigurement of the eye in six patients, phthisis bulbi in three patients, SO prevention in three patients, uncontrolled corneal ulcer in two patients, and painful blind eye in one patient.

The mean duration of follow-up was 30.9 ± 28.4 months (range: 3.1 months to 11.1 years). During the follow-up period, one patient out of 422 patients developed SO (0.24%). This patient did not undergo enucleation, and the VA of the traumatized eye was HM after corneal repair and IOFB removal. At 3 months post-injury, the VA of the sympathetized eye was 20/200, and the fundus revealed subretinal fluid at the papillomacular bundle; the patient was treated with intravenous methylprednisolone and methotrexate. Eleven years later, the VA of the sympathetized eye was maintained at 20/40–2.

### Risk factors for globe removal

Table 4 shows the univariable analysis for globe removal surgery. Lower raw OTS was significantly associated with globe removal. We used the reduced raw OTS for multivariable analysis. The reduced OTS was defined as the exclusion of the evaluation of RAPD during OTS calculation for all 422 patients. VA at presentation, assault injury, endophthalmitis, and panophthalmitis were significant risk factors for globe removal among patients with OGIs (Table 5).

**Table 4:** Univariable analysis of the risk factors for globe removal.

Variables	Globe removal		*p* value	Globe removal	*p* value (Wald-test)	*p* value (LR-test)
	No (n = 371)	Yes (n = 51)		Odds ratio (95% CI)		
*Gender*						
Male	346 (93.3)	44 (86.3)	0.077	1		
Female	25 (6.7)	7 (13.7)		2.20 (0.90, 5.39)	0.084	0.104
*Age*						
≤ 20 years	57 (15.4)	6 (11.8)	0.518	1		
> 20 to 40 years	137 (36.9)	24 (47.0)		1.66 (0.65, 4.29)	0.291	0.519
> 40 to 60 years	136 (36.7)	15 (29.4)		1.05 (0.39, 2.84)	0.927	
> 60 years	41 (11.0)	6 (11.8)		1.39 (0.42, 4.62)	0.591	
*Type of injury*						
Accident	355 (95.7)	46 (90.2)	0.091	1		
Assault	16 (4.3)	5 (9.8)		2.41 (0.84, 6.89)	0.100	0.126
*Presenting logMAR VA*						
Mean ± SD	1.82 ± 0.90	2.78 ± 0.24	< 0.001	283.28 (61.91, 1296.29)	< 0.001	< 0.001
Median (min, max)	2.3 (0, 3)	2.7 (1.9, 3)	< 0.001			
*BETT classification*						
Globe rupture	67 (18.1)	6 (11.8)	< 0.001	1^*a*^		
Penetrating	81 (21.8)	16 (31.4)		2.21 (0.82, 5.95)^*a*^	0.118	< 0.001
Perforating	6 (1.6)	7 (13.7)		13.03 (3.30, 51.45)^*b*^	< 0.001	
IOFB	217 (58.5)	22 (43.1)		1.13 (0.44, 2.91)^*a*^	0.797	
*IOFB*						
No	154 (41.5)	25 (49.0)	0.309	1		
Yes	217 (58.5)	26 (51.0)		0.74 (0.41, 1.33)	0.310	0.310
*Zone of injury*						
I	273 (73.6)	35 (68.6)	0.755	1		
II	62 (16.7)	10 (19.6)		1.26 (0.59, 2.68)	0.55	0.761
III	36 (9.7)	6 (11.8)		1.30 (0.51, 3.31)	0.58	
*RAPD*						
Negative	192 (51.8)	8 (15.7)	< 0.001	1^*a*^		
Positive	36 (9.7)	16 (31.4)		10.67 (4.25, 26.77)^*b*^	< 0.001	< 0.001
NA	143 (38.5)	27 (52.9)		4.53 (2.00, 10.27)^*c*^	< 0.001	
*Culture result*						
Negative	214 (74.3)	14 (32.6)	< 0.001	1		
Positive	74 (25.7)	29 (67.4)		5.99 (3.00, 11.95)	< 0.001	< 0.001
*OTS with documentation of the status of RAPD*						
Mean ± SD	65.37 ± 19.4	43.08 ± 12.87	< 0.001	0.92 (0.89, 0.95)	< 0.001	< 0.001
Median (min, max)	67 (19, 100)	43 (20, 70)	< 0.001			
*Reduced OTS calculation*						
Mean ± SD	67.01 ± 18.62	47.41 ± 12.21	< 0.001	0.93 (0.91, 0.96)	< 0.001	< 0.001
Median (min, max)	70 (19, 100)	43 (20, 70)	< 0.001			
*Uveal prolapse*						
No	298 (80.3)	34 (66.7)	0.026	1		
Yes	73 (19.7)	17 (33.3)		2.04 (1.08, 3.86)	0.028	0.033
*Maxillofacial fracture*						
No	366 (98.7)	48 (94.1)	0.026	1		
Yes	5 (1.3)	3 (5.9)		4.58 (1.06, 19.75)	0.042	0.062
*Globe infection*						
No	264 (71.1)	14 (27.5)	< 0.001	1^*a*^		
Endophthalmitis	106 (28.6)	14 (27.5)		2.49 (1.15, 5.40)^*b*^	0.021	< 0.001
Panophthalmitis	1 (0.3)	23 (45.0)		433.71 (54.56, 3447.51)^*c*^	< 0.001	
*Intraorbital foreign body*						
No	364 (98.1)	46 (90.2)	0.001	1		
Yes	7 (1.9)	5 (9.8)		5.65 (1.72, 18.54)	0.004	0.009
*Time lapse between injury and primary repair at our hospital*						
≤ 48 h	149 (72.0)	8 (80.0)	0.580	1		
> 48 h	58 (28.0)	2 (20.0)		0.64 (0.13, 3.11)	0.583	0.568

The *abc* values in columns not having a superscript in common differ significantly (*p* value < 0.05).

*CI* confidence interval; *LR* likelihood ratio; *logMAR* logarithm of the minimum angle of resolution; *VA* visual acuity; *BETT* Birmingham Eye Trauma Terminology; *IOFB* intraocular foreign body; *RAPD* relative afferent pupillary defect; *NA* not available; *OTS* Ocular Trauma Score.

**Table 5:** Multivariable analysis of the risk factors for globe removal.

Variables	Globe removal	*p* value (wald-test)	*p* value (LR-test)
	Adjusted odds ratio (95% CI)		
*Type of injury*			
Accident	1		
Assault	5.53 (1.29, 23.62)	0.021	0.026
*Presenting logMAR VA*	311.79 (42.99, 2261.49)	< 0.001	< 0.001
*Endophthalmitis or panophthalmitis*			
No	1^*a*^		
Endophthalmitis	3.58 (1.32, 9.67)^*b*^	0.012	< 0.001
Panophthalmitis*	734.94 (47.19, 11445.36)^*c*^	< 0.001	

The *abc* values in columns not having a superscript in common differ significantly (*p* value < 0.05).

*CI* confidence interval; *LR* likelihood ratio; *logMAR* logarithm of the minimum angle of resolution; *VA* visual acuity.

*Only one out of 24 patients with panophthalmitis did not undergo globe removal.

## Discussion

This study found that OGI occurred predominantly in males, with the peak incidence at 20 to 40 years and 40 to 60 years, which is consistent with the results of previous studies^3,4,17^. Among the patients with OGI, 75% were in the working age group, which is most likely to experience worked-related injury and be exposed to more dangerous activities. Among the patients with OGIs, 12.1% of the total patients underwent globe removal, primarily due to panophthalmitis. With the specific demographic distribution in this region, agriculture-related injury predominantly affected the characteristics of OGI, including a high prevalence of IOFB, endophthalmitis, and panophthalmitis.

Agriculture-related injury (54.7%) was a major cause of OGI and is more common than industry or workplace-related injury (20.4%). The most common mechanism of agriculture-related injury was injury caused by a flying object during mowing. It is associated with a high prevalence of IOFB (57.6%), compared with the prevalence of 32.9% reported by a study in Northern Thailand^2^ (with a range of 10.5% to 41% in other studies)^18,19^. This finding expresses the distinct characteristics of OGI in this region. Therefore, the use of safety eyewear and occupational safety protocols should be encouraged.

The mechanism of injury varied according to different age groups and settings. Assaults mostly affected middle-aged adult males. Surprisingly, age and gender were not independent predictors for globe removal. Assault injuries were typically severe; 16 out of 21 patients were assaulted by firearm and explosion. Thus, the chance of globe removal was higher at 23.8% as compared with that by accident. These data are also in agreement with a study by Bauza et al. that reported the rate of globe removal in case of assaults as 31.8%^20^. While the proportion of patients requiring global removal was high among those with perforating injuries, perforation did not retain its statistical significance in the multivariable model, mainly owing to the poor VA in all patients with perforation.

The key finding in our study was that the presenting VA was a powerful variable to identify in patients who feasibly underwent globe removal. An increase of 0.1 logMAR VA affected the possibility for globe removal surgery by 31.2-fold. The authors suggest that in patients with trauma, the presenting VA can help physicians in counseling patients about their chance of globe loss. The percentage of globe removal was high in NLP eyes. However, the presence of an eye with NLP is not an indication for enucleation to prevent SO since the rate of SO is low. The estimated overall incidence proportion of SO following OGI was 0.19%^21^. Only one patient in this study developed SO; however, it was controlled with immunosuppressive drugs.

This study found that the ratio of culture-negative intraocular infection was higher in the non-globe removal group compared with that in the globe removal group, similar to the results of a study by Vedantham et al.^22^, wherein culture-negative traumatic endophthalmitis was associated with better visual outcome. However, the objective of that study, the visual outcome, was different from our study. The rate of enucleation in eyes infected with *Pseudomonas* and *Bacillus* species was higher than that in those infected with *Staphylococcus* species, corresponding to the results of a previous study^23^, in which half of the eyes infected by *Bacillus* or *Pseudomonas* species underwent enucleation.

The possibility of injury with contaminated IOFB was high in agriculture areas. Therefore, penetrating injury, IOFB, and perforating injury were significantly associated with globe removal in the univariable analysis, compared with that with a ruptured globe. This finding was contrary to the results of studies from developed countries. The most common organism associated with globe removal was the *Bacillus* species, which caused rapidly progressing intraocular infection following traumatic injuries. *Bacillus cereus* exhibits robust bacterial replication, migration throughout the eye, and toxin production^24^. Most patients with globe contamination due to *Bacillus cereus* may develop acute endophthalmitis as soon as within a few hours of injury. Therefore, the window of opportunity for therapeutic intervention in *Bacillus cereus* endophthalmitis is short compared with that of other pathogens^24^.

OTS has been recommended as a valuable tool for the classification of OGIs and the determination of the final VA. Our data showed that OTS was a significant predictor for globe removal surgeries in univariable analysis. Brundridge et al.^11^ reported that a lower OTS resulted in a higher likelihood of enucleation. Savar et al.^7^ showed that only 22% of patients in OTS category 1 underwent enucleation. However, OTS aims to predict the final visual prognosis and is not designed to predict enucleation^16^. In our study, multivariable analysis demonstrated that OTS was not a significant variable. Additionally, the status of RAPD, one of the variables used to calculate OTS, was not well documented. This study showed that almost half of all patients with OGIs did not have a recorded RAPD, which could probably not be assessed due to very severe ocular injuries, uncooperativeness of the patient, and clinical illness. Schörkhuber et al.^25^ reported that OTS calculation without evaluating RAPD was easily applicable while remaining significantly prognostic for visual outcome. Therefore, the use of OTS category with or without the evaluation of RAPD to predict the possibility of globe removal surgeries still requires further study.

Endophthalmitis or panophthalmitis significantly increases the risk of globe removal. The rate of endophthalmitis or panophthalmitis among patients with OGIs was 50% in our study, compared with 0–16.5% in previous studies^19,23,26^. More than 95% of patients with panophthalmitis underwent globe removal. In addition, this finding showed a high prevalence of severe endophthalmitis and panophthalmitis. Access to a health center may have been delayed by inconvenient transportation and lack of public health knowledge. The rate of endophthalmitis or panophthalmitis in our study was higher than that in previous studies; however, the rate of globe removal surgery was similar to that reported by previous studies^7,8^, indicating that it may be due to effective treatment against microorganisms. Hence, the prevention of endophthalmitis or panophthalmitis in cases of OGI is crucial for the preservation of the vision and the globe. Immediate globe repair with intravitreal antibiotics and additional broad-spectrum systemic antibiotics reduces the devastating complications of OGI.

Panophthalmitis has been identified as a risk factor with a 200-fold increased risk for globe removal than that with endophthalmitis. The other reason that OTS could not predict the potentiality of globe removal was because we calculated OTS of panophthalmitis using the score of endophthalmitis. Thus, the severity of panophthalmitis is more extensive than endophthalmitis, and the main treatment is enucleation.

The limitations of this study include its retrospective design and the lack of pertinent information for several patients, such as the use of protective eyewear and the evaluation of RAPD. For the bilateral OGIs, only one eye was selected to be part of the data analysis which was a possible cause of bias; however, we used the computer program randomization to eliminate an allocation bias. This study collected medical records from a single tertiary care center only; this may not have covered all minor injuries in this region. Multicenter prospective studies with a large number of enrolled patients or national surveys need to be conducted. The age groups and mechanisms of injuries in our study were diverse; further studies should focus on specific populations, such as the pediatric age group, work-related injuries, or assaults, to identify the risk factors for globe removal more specifically.

In conclusion, our study showed that assault injury, presenting VA, and endophthalmitis or panophthalmitis were the significant predictive factors associated with globe removal. This information may help in counseling patients and their families about the possibility of globe removal. These data also advocate the use of protective eyewear and a national health policy for workers in agricultural areas.

## Data Availability

All unidentifiable data are available from the corresponding author upon reasonable request.

## References

[1] Négrel AD, Thylefors B (1998). The global impact of eye injuries. Ophthalmic. Epidemiol..

[2] Supreeyathitikul P (2020). Epidemiology and outcomes following open globe injury in agricultural region, an 11-year experience. Ophthalmic. Epidemiol..

[3] Beshay N (2017). The epidemiology of open globe injuries presenting to a tertiary referral eye hospital in Australia. Injury.

[4] Okamoto Y (2019). Clinical characteristics and outcomes of open globe injuries in Japan. Jpn. J. Ophthalmol..

[5] Ababneh OH, AboTaleb EA, Abu Ameerh MA, Yousef YA (2015). Enucleation and evisceration at a tertiary care hospital in a developing country. BMC. Ophthalmol..

[6] Zhang Y, Zhang MN, Wang X, Chen XF (2015). Removal of the eye in a tertiary care center of China: A retrospective study on 573 cases in 20 years. Int. J. Ophthalmol..

[7] Savar A, Andreoli MT, Kloek CE, Andreoli CM (2009). Enucleation for open globe injury. Am. J. Ophthalmol..

[8] Dunn ES, Jaeger EA, Jeffers JB, Freitag SK (1992). The epidemiology of ruptured globes. Ann. Ophthalmol..

[9] Sobaci G, Akýn T, Mutlu FM, Karagül S, Bayraktaret MZ (2005). Terror-related open-globe injuries: A 10-year review. Am. J. Ophthalmol..

[10] Thach AB (2008). Severe eye injuries in the war in Iraq, 2003–2005. Ophthalmology.

[11] Brundridge W (2009). Open globe trauma in a military hospital: A review of the ocular trauma score to help predict enucleation or evisceration. Graefes Arch. Clin. Exp. Ophthalmol..

[12] Kuhn F (1996). A standardized classification of ocular trauma. Ophthalmology.

[13] Kuhn F, Morris R, Witherspoon CD, Mester V (2004). The birmingham eye trauma terminology system (BETT). J. Fr. Ophtalmol..

[14] Schulze-Bonsel K, Feltgen N, Burau H, Hansen B, Bach M (2006). Visual acuities "hand motion" and "counting fingers" can be quantified with the freiburg visual acuity test. Invest. Ophthalmol. Vis. Sci..

[15] Lange C, Feltgen N, Junker B, Schulze-Bonsel K, Bach M (2009). Resolving the clinical acuity categories "hand motion" and "counting fingers" using the Freiburg Visual Acuity Test (FrACT). Graefes Arch. Clin. Exp. Ophthalmol..

[16] Kuhn F (2002). The ocular trauma score (OTS). Ophthalmol. Clin. North Am..

[17] Fujikawa A (2018). Visual outcomes and prognostic factors in open-globe injuries. BMC. Ophthalmol..

[18] Loporchio D (2016). Intraocular foreign bodies: A review. Surv. Ophthalmol..

[19] Ojuok E (2021). Predictive factors of enucleation after open globe injuries. Graefes Arch. Clin. Exp. Ophthalmol..

[20] Bauza AM (2013). A 10-year review of assault-related open-globe injuries at an urban hospital. Graefes Arch. Clin. Exp. Ophthalmol..

[21] He B (2022). The incidence of sympathetic ophthalmia after trauma: A meta-analysis. Am. J. Ophthalmol..

[22] Vedantham V, Nirmalan PK, Ramasamy K, Prakash K, Namperumalsamyet P (2006). Clinico-microbiological profile and visual outcomes of post-traumatic endophthalmitis at a tertiary eye care center in South India. Indian J. Ophthalmol..

[23] Bhagat N, Nagori S, Zarbin M (2011). Post-traumatic infectious endophthalmitis. Surv. Ophthalmol..

[24] Mursalin MH, Livingston ET, Callegan MC (2020). The cereus matter of Bacillus endophthalmitis. Exp. Eye. Res..

[25] Schörkhuber MM, Wackernagel W, Riedl R, Schneider MR, Wedrich A (2014). Ocular trauma scores in paediatric open globe injuries. Br. J. Ophthalmol..

[26] Ahmed Y, Schimel AM, Pathengay A, Colyer MH, Flynn HW (2012). Endophthalmitis following open-globe injuries. Eye. (Lond).

